# Anatomical Drawings

**Published:** 1850-10

**Authors:** 


### ANATOMICAL DRAWINGS.

We regard good anatomical drawings as the means nearest the excellence of actual dissections, for imparting sound and valuable knowledge to students of medicine, and in the term student we do not mean merely the neophyte in the approaches to the temple, but all those who, after attaining high distinctions in all the departments of medicine, continue to press onward and upward in the pursuit of knowledge. We have been reminded of the value of anatomical drawings, by a recent examination of several very superior specimens, drawn and colored by Mr. Charles Wolford, a very tasteful and skillful artist of this city. These specimens were prepared for Dr. Gross, and Professor Miller has, in process of execution, some beautiful drawings, for his department.

They are executed in handsome style, and cannot fail to give accurate and impressive anatomical information to the Professor's classes. The artist deserves much praise for the admirable manner in which he has accomplished the orders of his medical employers, and we hope his useful art will not be suffered to languish for the want of patronage. A series of enlarged drawings of Maclise's anatomical plates of the anatomy of hernia, would be invaluable in a course of surgical teaching, for it would give students a much more accurate idea of the anatomy of hernia, than is imparted by any process of didactic teaching we are acquainted with. B.

